# Correction: ‘Voice needs teeth to have bite’! Expanding community-led multisectoral action-learning to address alcohol and drug abuse in rural South Africa

**DOI:** 10.1371/journal.pgph.0002187

**Published:** 2023-07-12

**Authors:** Lucia D’Ambruoso, Denny Mabetha, Rhian Twine, Maria van der Merwe, Jennifer Hove, Gerhard Goosen, Jerry Sigudla, Sophie Witter, On behalf of the Verbal Autopsy with Participatory Action Research (VAPAR)/Wits/Mpumalanga Department of Health Learning Platform

There are errors in the article Abstract. The correct Abstract is: There is limited operational understanding of multisectoral action in health inclusive of communities as active change agents. The objectives were to: (a) develop community-led action-learning, advancing multisectoral responses for local public health problems; and (b) derive transferrable learning. Participants representing communities, government departments and non-governmental organisations in a rural district in South Africa co-designed the process. Participants identified and problematised local health concerns, coproduced and collectively analysed data, developed and implemented local action, and reflected on and refined the process. Project data were analysed to understand how to expand community-led action across sectors. Community actors identified alcohol and other drug (AOD) abuse as a major problem locally, and generated evidence depicting a self-sustaining problem, destructive of communities and disproportionately affecting children and young people. Community and government actors then developed action plans to rebuild community control over AOD harms. Implementation underscored community commitment, but also revealed organisational challenges and highlighted the importance of coordination with government reforms. While the action plan was only partially achieved, new relationships and collective capabilities were built, and the process was recommended for integration into district health planning and review. We created spaces engaging otherwise disconnected stakeholders to build dialogue, evidence, and action. Engagement needed time, space, and a sensitive, inclusive approach. Regular engagement helped develop collaborative mindsets. Credible, actionable information supported engagement. Collectively reflecting on and adapting the process supported aligning to local systems priorities and enabled uptake. The process made gains raising community ‘voice’ and initiating dialogue with the authorities, giving the voice ‘teeth’. Achieving ‘bite’, however, requires longer-term engagement, formal and sustained connections to the system. Sustaining in highly fluid contexts and connecting to higher levels are likely to be challenging. Regular learning spaces can support development of collaborative mindsets in organisational contexts aligning community voice with state capacity to respond.
