# Supplementary material for: ‘Voice needs teeth to have bite’! Expanding community-led multisectoral action-learning to address alcohol and drug abuse in rural South Africa
Source: PLOS Glob Public Health. 2022 Oct 19;2(10):e0000323. doi: 10.1371/journal.pgph.0000323 (PMC10022044; doi:10.1371/journal.pgph.0000323)
Supplement: S1 Table — (DOCX) [file pgph.0000323.s001.docx]

S1 Table: Government and community stakeholder analysis of AOD abuse in rural communities

| Goal | Reduce AOD abuse in rural communities |
| --- | --- |
| Barriers | Solutions |
| - Taverns: Trading hours, licensing, access control | - Law enforcement: police service implement acts and regulations - Visible policing in rural communities to support compliance with e.g., opening hours |
| - Home: Unemployment, hunger, dysfunctional families, abuse, poverty | - Establish community-based social support groups - Community level partnerships focussed on social disadvantage and AOD abuse |
| - Schools: Intimidation, poor performance, lack of resources, lack of integration | - Public awareness campaign on AOD abuse as a local public health issue |
| - Clinics: Lack of screening and health professional incapacity | - Supply screening tool to identify individuals at risk - WBPHCOTs to support and assist in screening |
| - Hospitals: Under resourced and lack of intervention | - Rehabilitation centres based in communities to support health service |
| - SAPS: Bribery, lack of prioritisation and under resourcing | - Intersectoral collaboration to support improved working practices and address lack of resourcing - Monitor adherence to legal prescripts, public awareness on legal framework |
| - DSD: Demand of social grant, burden at community level, lack of screening | - Establish support groups at community level with a focus on awareness raising round social support and prevention and education on AOD abuse |
| Obstacles to effective policy implementation: | |
| - Practical policies: practicality of most policies may not be sufficient - Shortages of resources: in practice, resource shortages make some policies un-implementable - Donor driven policies problematic: most policies are funded by external organizations which gives implementers less autonomy. Consequentially, policy usually does not address the problems that it seeks to address - Policy ‘noise’: policies are updated frequently, and often before the initial policy has been fully implemented and evaluated - Inconsistent messaging: teaching different messages for same policy that addresses same health problem is an issue. For example, Soul City with *Phuza Wise* and *Kimoja* have different messages, which is confusing to the public | |

DSD: Department for Social Development; SAPS: South African Police Service; WBPHCOT: Ward-Based Primary Health Care Outreach Team
