# Supplementary material for: ‘Voice needs teeth to have bite’! Expanding community-led multisectoral action-learning to address alcohol and drug abuse in rural South Africa
Source: PLOS Glob Public Health. 2022 Oct 19;2(10):e0000323. doi: 10.1371/journal.pgph.0000323 (PMC10022044; doi:10.1371/journal.pgph.0000323)
Supplement: S2 Table — (DOCX) [file pgph.0000323.s002.docx]

S2 Table: Governmental and community stakeholder analysis of existing policies, implementing partners and recommendations to address AOD abuse

| Existing policies and programmes | Implementing partners |
| --- | --- |
| DSD   - Prevention and Treatment of Substance Abuse Treatment Act and National Drug Master Plan (reviews of these policies currently underway) - Provincial Drug Master Plan adapted from the national policy to meet provincial needs - Mpumalanga Liquor Licensing Act to regulate liquor stores - Family preservation which includes parenting support programme - Child Justice Act - Kimoja *I’m fine without drugs* drug prevention programme (in schools) joined with She Conquers to encourage youth to live better lifestyle without drugs | Globally funded programmes, USAID/ PEPFAR addressing the impact of alcohol on prevention, care, and treatment of HIV in Southern and Eastern Africa. The programme aims to develop strategies that address issues related to alcohol intake and rate of HIV. |
| DBE   - Schools safety and drug abuse strategy (under the Provincial Drug Master Plan) - Peer education on social skills and risky behaviour (pilots) | Non-Profit Organisations (NPOs): SANCA, Treatment and Rehabilitations Centres |
| DoH   - Five components of Health Lifestyles - Mini Drug Master Plan | Non-Profit Organisations (NPOs): SANCA, Department of Basic Education, Department of Social Development, Research institutions |
| National Development Plan (NDP) 2030   - Policy aims to combat poverty among South Africans promoting gender equality and creating employment and education opportunities for young people | DPWRT Expanded Public Works Programme (EPWP): aims to address poverty and provide income relief through temporary work for unemployed people and make sure that youth get appropriate skills training and employment |
| Insights and recommendations | |
| - *Integrating approaches*: services should be provided through integrated approaches with collaboration among the provincial substance abuse program, Local Drugs Action Committees and integrated school safety forums working together with WBPHCOTs to reduce drugs and alcohol rate among youth; - *Regulate alcohol distribution (taverns):* there is a need to change the current alcohol businesses licensing and tavern proliferation e.g., through advocacy by presenting evidence collected in villages about consequences of drugs and alcohol abuse. Traditional structures may also be involved e.g., since there are high rates of alcohol abuse among youth during youth initiation ceremonies, along with WBPHCOTs; - *Providing alternatives for young people*: Training available, but employment difficulties remain: youth do get training but they are often not employed after internships due to lack of vacant posts which usually discourage the others to seek and receive the same training. | |

DBE: Department of Basic Education; DSD: Department for Social Development; DPWRT: Department of Public Works, Roads and Transport; SANCA: South African National Council on Alcoholism and Drug Dependence
