# Supplementary material for: ‘Voice needs teeth to have bite’! Expanding community-led multisectoral action-learning to address alcohol and drug abuse in rural South Africa
Source: PLOS Glob Public Health. 2022 Oct 19;2(10):e0000323. doi: 10.1371/journal.pgph.0000323 (PMC10022044; doi:10.1371/journal.pgph.0000323)
Supplement: S3 Table — (DOCX) [file pgph.0000323.s003.docx]

S3 Table: Local Action Plan to address AOD abuse among youth and adolescents

| Action/lead actors | Lead and responsible actors | Baseline | Target | Measure |
| --- | --- | --- | --- | --- |
| Community stakeholders | | | | |
| 1. Identify hot spots for substance abuse: call community leaders in Agincourt HDSS study area together to discuss AOD as a priority public health problem and identify liquor outlets operating illegally and drug outlets that are often hidden | Community stakeholders led by the youth group (lead) with support from HDSS; Non-Government Organisations; South African National Council on Alcoholism and Drug Dependence; LoveLife; Home Based Care workers | Anecdotal knowledge but not an official list | One ‘official’ list | List of ‘hotspots’ and of signs of where people are able to buy drugs |
| 2. Strengthen collaboration between traditional leaders and law enforcement (SAPS), local municipalities, the former liquor board, now Mpumalanga Economic Regulator, to reduce noncompliant outlets with an initial focus on closing hours | Community stakeholders led by CDF member at community level (lead) with support from Local Drug Action Committee; Mpumalanga Economic Regulators | Good relationships between law enforcement and traditional leaders but poor compliance from liquor outlets | Improved compliance by liquor outlets (longer-term goal) | Follow up conversation on role clarification to initiate strengthening collaboration |
| Department of Health |  |  |  |  |
| 3. Encourage professional nurses to adopt primary or secondary school to support, provide information on drug and substance abuse and to be *on the phone* for emergencies in junior schools | Department of Health MCWYH coordinator at district level (lead),  Department of Social Development Local Municipality; South African Police Services; Department of Cooperative Governance and Traditional Affairs ; Department of Culture, Sports and Recreation | Unknown most schools have a health support team | Each nurse visits their school quarterly | Report from nurses on adoption, support, uptake and emergencies |
| Other government departments and agencies | | | | |
| 4. Advocate for aligned application of legislation between SAPS and Department of Justice (DOJ) concerning substance abuse law enforcement in liquor outlets | South African Police Service Member of the local drug action team (lead), Department of Justice;  (province); National Prosecuting Authority | Under 1% conviction rates | Double conviction rate (2 years plus) | Reports and group advocacy; Conviction rates for drug related cases; Non-compliance with regard to liquor outlets |
| 5. Disseminate information on roles and responsibilities of government departments, collectively or individually, for AOD abuse. October 2018 is social development month and there are DSD awareness raising activities planned (‘Blitz’) | Department of Social Development Social worker working in the sub-district (lead), Local Municipality; Department of Culture, Sports and Recreation; Department of Health; South African Police Services; Department of Cooperative Governance and Traditional Affairs; Department of Community Safety, Security And Liaison; South African National Council on Alcoholism and Drug Dependence; Non-Government Organisations Local Drugs Action Committee; Community stakeholders | None | 1 event | One cluster in sub-district. Follow up conversation during social development month |
| 6. Mobilise resources for community-based treatment centre for drug and substance abuse: established by and owned at community level, supported by departments (doctors and social workers) and NGOs | Department of Social Development Social worker working in the sub-district (lead), Community stakeholders; Department of Health; Local Municipality; Traditional leaders; Non-Governmental Organisations; Faith-Based Organisations (Churches) | None | 1 fully functional community-based treatment centre in the sub-district | Organisation who will start this will be identified. Longer term 3-5 years for fully functional centre |
