# Supplementary material for: ‘Voice needs teeth to have bite’! Expanding community-led multisectoral action-learning to address alcohol and drug abuse in rural South Africa
Source: PLOS Glob Public Health. 2022 Oct 19;2(10):e0000323. doi: 10.1371/journal.pgph.0000323 (PMC10022044; doi:10.1371/journal.pgph.0000323)

S1 Text: Department of Health Research Brief

****DEPARTMENT OF HEALTH **RESEARCH BRIEF**

Verbal Autopsy with Participatory Action Research (VAPAR)

Expanding the knowledge base through partnerships for action on health equity

*Series 1, Number 4, January 2018*

**“It destroys families, communities and futures”:**

local knowledge to reduce alcohol and drug abuse

**Alcohol and other drug (AOD) abuse is a growing problem in South Africa. Consumption is generally hazardous, associated with risky sexual behaviours, crime, violence, adverse effects on health behaviours, care seeking, utilisation and treatment adherence, with implications for HIV infection, transmission, and conditions including heart disease, cancers, stroke and seizures. Working in partnership with local communities, this research aimed to understand the health and social impacts of, and local priorities to address, AOD abuse in Mpumalanga.**


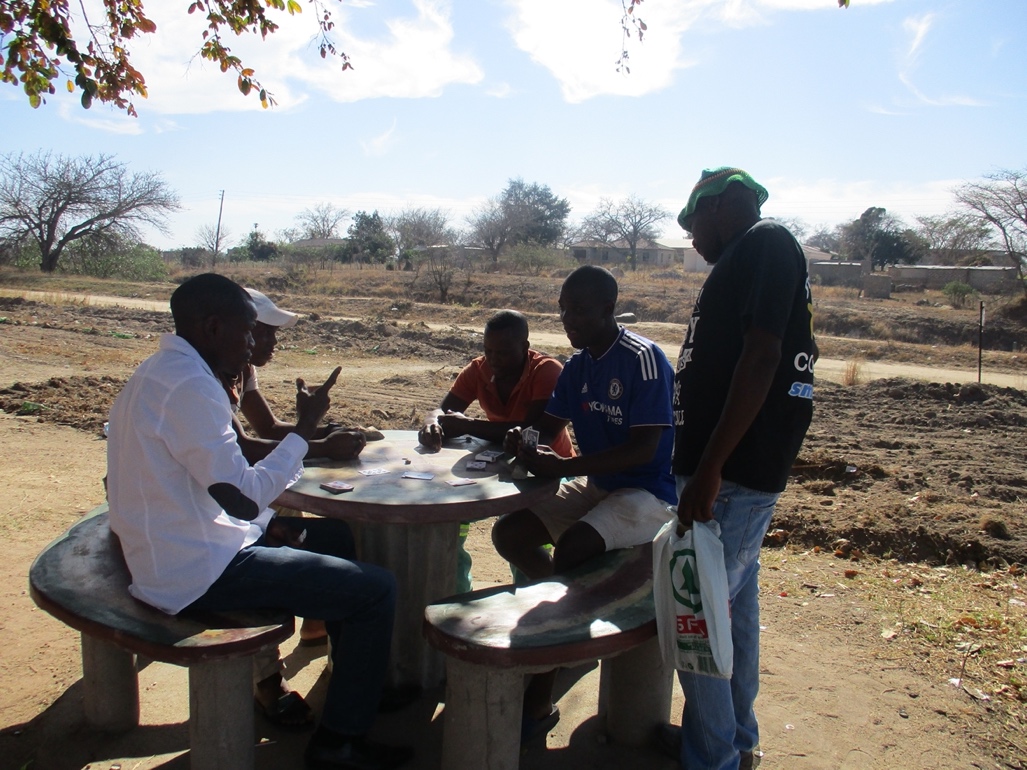


*Increasing numbers of taverns reported*

AOD abuse has many effects on health, wellbeing, society and the economy. The issue is preventable and predominately effects socially disadvantaged groups. Routine mortality data from Agincourt HDSS was analysed and combined with community research, where we worked with adult, elder and youth participants. We also developed visual evidence with community participants to complement statistical data.

**Drivers of AOD abuse**

- ***Taverns***: Large and increasing number of taverns with long opening hours (some 24 hours), gambling and dirty environments reported. Taverns reported to influence and enable AOD abuse, unsafe sex, prostitution, violence and drug taking. Children smoking/drinking at young ages due in part to accessibility via taverns.

*“Yesterday I saw blood flowing like water at the tavern”* ***Community participant***

- ***Poverty:*** Multiple structural influences: poverty, poor education and low employment. Some children cannot take the stress from being poor and use AOD to relieve stress.
- ***Modern culture:*** Adult/elder participants noted TV, media and peer pressure as drivers, noting modern behaviours do not align with Shangaan culture as modest and prioritising respect for others and self. Youth participants’ views were very different, referring to the need for social/peer support stating: “not abusing AOD is dehumanising”.

*“People selling alcohol serve no purpose… If we had power, we wouldn’t have taverns in this community”* ***Community participant***

- ***Substance abuse***: Widespread use of marijuana, benzene and glue described. Reports that ARVs are mixed and smoked, called ‘Nyaupe’, often among children.
- ***Traditional practices***: Several reported e.g. traditional beer (Xipayoni).
- ***Corruption:*** drug dealers reportedly collaborate with police and community leaders.
- ***Adults/elder distress and anxiety:*** significant distress and anxiety due to behaviours related to AOD abuse, e.g. theft within the household, and sexual assault on relatives.
- ***Lack of planning and leadership:*** in households, communities and the authorities and lack of information, rehabilitation services and recreational facilities for youth.

**Impacts of AOD**

**Deaths linked to drug/alcohol abuse (Agincourt HDSS 2014-15)**

Road traffic accident 42 ***

Self-harm/suicide 22 ***

Assault 21 ***

HIV-related 185 **

Lung cancer 36 **

Liver damage 24 **

Other transport accident 17 **

Other injury 4 **

TB 104 *

Stomach cancer 70 *

Heart attack 47 *

Heart disease 44 *

TOTAL 513 **

* May be related to substance abuse

** Likely to be linked to substance abuse

*** Very likely to be linked to substance abuse

- ***Behavioural:*** many reported: crime to support addiction, drunk driving, gambling, poor decision-making, risky behaviours, unsafe sex, prostitution, loss of dignity, poor medication compliance, violence, road traffic accidents, poor nutrition, self-care and crime.
- ***Health***: many reported: assault (including sexual assault), cancer, disability, HIV/AIDS, injuries (including RTAs), malnutrition, mental health, overdose, stress, stroke, suicide, TB, heart, lung, liver and other vital organ conditions and unplanned pregnancy.
- ***Social:*** immediate and long-term impacts on education, employment and ability to lead healthy and productive lives, crime and prison.
- ***Destroys future, destroys communities***: Considering the collective effects, AOD abuse described as wholly destructive of families and communities.

**Statistical data 2014-15**

- Of 1,524 deaths >500 may have been due to/influenced by, AOD abuse.

**Actions recommended**

***Reduce rates of AOD abuse***: Communities developed integrated action plans, inclusive of multi-sectoral partnerships and monitoring mechanisms to achieve realistic and achievable reductions in rates of AOD abuse as follows:

- ***Regulate taverns:*** reduce opening hours, reduce numbers of taverns, enforce age restrictions, provide fewer youth attractions in taverns (e.g. pool tables), more police presence in taverns, regular inspections of taverns and community patrols, stronger community regulation of taverns including community police forum (CPF) involved in tavern regulation, Indunas to work with owners to improve relationships with communities.
- ***Education and employment:*** Registers to monitor attendance, searches and drug tests in schools, co-parenting between parents and educators. Improve opportunities after matric (e.g. bursaries). Significant employment opportunities via community education, rehabilitation and recreation, local garden farming initiatives etc. (see below). In other settings, NGOs and social enterprises address social needs via service provision delivered through “supported employment and volunteering” to break cycles of addiction and poverty.
- ***Community rehab and drop-in:*** improved referral to existing support services, and building of new rehab facilities, youth drop-in recreation centres with authorities (e.g. Depts. of Health, Education and Social Development, National Council SANCA) and NGOs.
- ***Community health education:*** educating people on AOD abuse in schools and clinics critical. Youth reportedly engage in AOD due to lack of guidance/financial support. More teachers, social workers, emotional support, food aid and housing support for people living in poverty.
- ***Community partnerships:*** Indunas with more powers to head AOD action partnerships with ward committees, parents***,*** CPF, community development forum (CDF), pastors, magistrates, community health workers, schools. Councillors to work effectively for community.
- ***Research:*** continuing to collaborate with Wits to understand the burden of avoidable mortality owing to AOD.

**
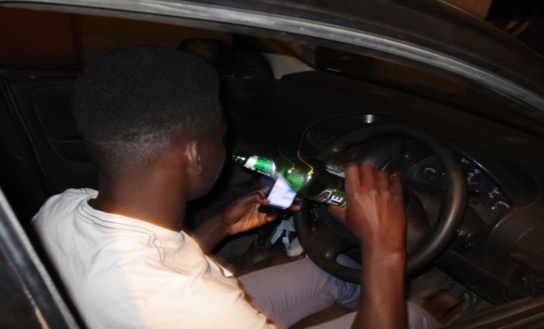

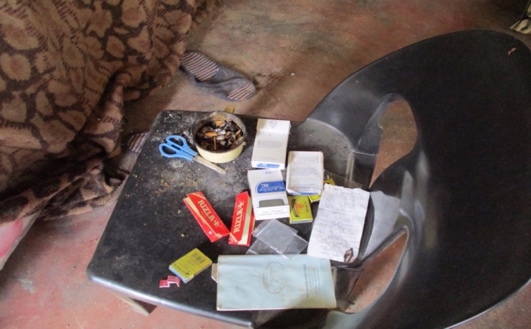

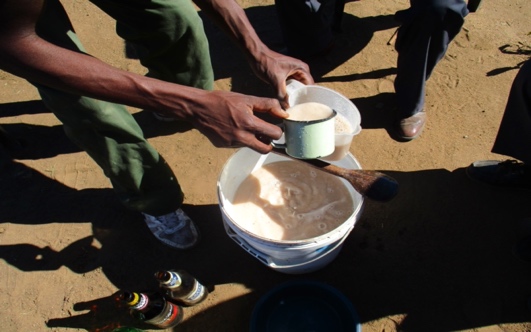
Visual evidence**

*Drunk driving in youth*

*Traditional beer*

*Marijuana use*

Verbal Autopsy with Participatory Action Research (VAPAR) is a programme of partnerships research funded the Health Systems Research Initiative from Department for International Development (DFID)/Medical Research Council (MRC)/Wellcome Trust/Economic and Social Research Council (ESRC) (MR/N005597/1) and MR/P014844/1).

Image credits: © VAPAR 2017 Permissions have been secured for the reproduction of images.


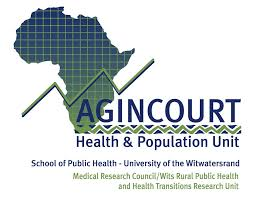

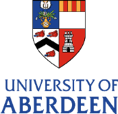

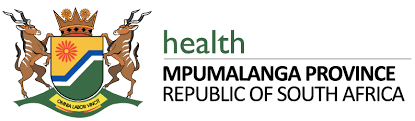

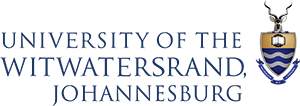

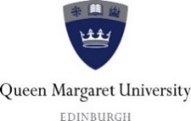

Supplement: S1 Text — (DOCX) [file pgph.0000323.s004.docx]
