# Supplementary material for: ‘Voice needs teeth to have bite’! Expanding community-led multisectoral action-learning to address alcohol and drug abuse in rural South Africa
Source: PLOS Glob Public Health. 2022 Oct 19;2(10):e0000323. doi: 10.1371/journal.pgph.0000323 (PMC10022044; doi:10.1371/journal.pgph.0000323)
Supplement: S2 Text — (DOCX) [file pgph.0000323.s005.docx]

S2 Text: Local Action Plan Monitoring Proforma


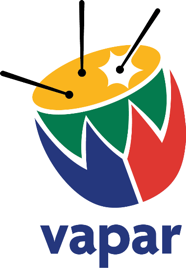
**Verbal Autopsy with Participatory Action Research (VAPAR):**

**Expanding the knowledge base through partnerships for action on health equity**

Local Action Plan: MONITORING PROFORMA Version 20.11.2018

Youth and adolescent health related to alcohol and other drug abuse /

Child health related to lack of clean, safe water

Guideline: This proforma should be used to capture both stated and unanticipated/ad hoc actions arising from the VAPAR process. For the former, complete the action in the table below verbatim from the local action plan (LAP). For the latter, strikethrough the LAP excerpt table and complete sections 1-5

LAP Excerpt

| **Action** | **Actors** | **When** | **Measure** | **Baseline** | **Target** | **Notes** |
| --- | --- | --- | --- | --- | --- | --- |
|  |  |  |  |  |  |  |

Action report

| 1 Action: |  |
| --- | --- |
| 2 Date: |  |
| 3 Update (may not be necessary for ad hoc actions): |  |
| 4 Supporting evidence (including any follow up): | [Joint account Researcher/Stakeholder - enter max 200 words, agreed with Stakeholder]  (This is based on our observation) |
|  | ……………………………………………………….. Stakeholder |
|  | ……………………………………………………….. Wits |
| 5 Notes | [Researcher account - enter max. 300 words on   1. Interaction with stakeholder: 2. Substantive observations on action progression: 3. learning e.g. roles and functions of groups/individuals]: |
| 6 Any relevant documents |  |
