# Supplementary material for: ‘Voice needs teeth to have bite’! Expanding community-led multisectoral action-learning to address alcohol and drug abuse in rural South Africa
Source: PLOS Glob Public Health. 2022 Oct 19;2(10):e0000323. doi: 10.1371/journal.pgph.0000323 (PMC10022044; doi:10.1371/journal.pgph.0000323)
Supplement: Attachment — Submitted filename: Response to Review.docx [file pgph.0000323.s006.docx]

| Reviewer(s)' Comments to Author(s): | Author response | Page |
| --- | --- | --- |
| Reviewer #1: |  |  |
| The topic and research process are very interesting, being a good example of participative process to solve health problems highlighting the normal limitations of this type of initiatives.  I provide some suggestions mostly focus to improve the presentation of the manuscript | The authors thank Reviewer 1 for insightful comments. | - |
| Results  While reading the results are difficult to follow. The researchers mention as themes:  a) dynamics of the process.  b) actor interactions and interfaces.  c) impacts and degrees of impacts.  d) enabling and constraining contextual factors.  Nonetheless, the results do not highlight the above, presenting other subtitles that promote confusion. Also, the results do not include quotes or evidence of the description presented, the researchers present observation of actor’s dynamics, combined with impressions and actions of the research team and theoretical arguments of the methodology. I suggest revise and restructure this section.  I suggest including:  - A summary of the findings from the VA to understand the magnitude of the problem in the manuscript going beyond the 30% of prevalence of the problem. This allows a better understanding of the resources considered by the different actors.  - A table with the principal findings related to PAR in every phase.  - A Figure (or more if is needed) that represent the real action-learning process that resulted from the implementation of PAR. This allows to respond in a clear way to the main objective of the research. | In the revised manuscript, the results include clearer and more explicit reference to the categories derived from analytical frameworks. We have woven these references throughout the results to anchor more clearly and directly the analysis to the frameworks that organise it. We have also further supplemented the Results with narrative data.  Regarding a summary of the VA results. We have provided the Research briefing output as Supporting Information detailing this analysis.  We have revised Figure 1 to provide additional information that summarises the principal findings related to each phase of the process. The analysis of VA data to understand attributable burden of AOD-related mortality is situated within this account.  Figure 1 also contains a summary of the mechanisms and outputs related to every phase. | 11-18, Supporting Information 1, Fig. 1 |
| Discussion  “Derive transferrable learning” is the second objective, I suggest moving it from the discussion to the results section.  If the quote placed in the discussion is not really needed, I suggest to eliminate.  I suggest adding a paragraph that present the discussion points that will be develop in this section, I encourage authors to present it in a structured way for a more friendly reading. | The authors thank Reviewer 1 for this very useful comment. The content in the Discussion section on transferrable learning has been relocated in the Results section.  The quotation provided in the discussion is intended to help illustrate the ‘intrinsic and instrumental’ value of accountability processes. Moreover, and reflecting on Reviewer 2’s supportive remarks on the use of this quotation (acknowledging it is lengthy) the authors have opted to retain this. However, we have edited the quotation in response to this comment.  A paragraph has also been included at the start of the discussion section, to reflect the content that will be presented in this section of the paper. | 11-18  19-23 |
| References  42% of the reference have more than 5 years. I suggest updating some of them. | The references have been thoroughly updated to reflect more recent evidence, methodological developments and debates. Over 60% of the references are now made to articles published within the last 5 years. | 23-31 |
| Reviewer #2: |  |  |
| This paper opens a window into the experience of drawing together rural community stakeholders to examine and address the local public health crisis of AOD deaths in South Africa via action-learning processes with diligence and honesty. Processes are recounted systematically so that the reader feels immersed in the many, well-planned research stages and can understand the strategies and outcome goals embedded at every stage of presentation.  Clearly the research team’s long experience in participatory methods and study design come through with ease so that the reader can derive much from this well-written formal accounting of the intervention. | Thank you for the encouraging comments and positive feedback. | - |
| My comments are therefore offered to expand the context of study particulars study so as to distinguish it from any comparable action-learning experience. Because the researchers are so immersed, and so knowledgeable of the rural community public health field--its structures (internal and vis-à-vis local and national governing authorities), culture, deficiencies, and on-going resource challenges—the paper overall assumes conveys a very process-oriented tone, though at the risk of losing a sense of societal particulars which make the AOD crisis both personal and poignant within South Africa’s countryside.  This problem of context can be easily resolved through the inclusion of more anonymized participant quotes. As it stands, only three quotes are presented, and one at the very end rather than in the results section where one expects to find them. Participant quotes are the heart of qualitative thematic data and convey what figures and prose cannot. | The authors thank the reviewer for encouraging us to bring through these contextual features. The revised manuscript contains a fuller account of the societal roots and impacts of the burden of AOD abuse. In the Results section, we have also provided more narrative quotes. | 3-5  11-18 |
| A second related issue is that the dimensions of the AOD crisis are described late in the paper as part of the discussion. In fact, their appropriateness is wanting in the paper’s Introduction to set the tone of urgency currently missing as one reads sections of recruitment, report drafting, and analysis so meticulously described. As is, it feels like there is an excessive detachment from the underlying health problem whose recognition is being process-driven.  Nonetheless, I recommend this paper for publication with minor revisions described in detail below.  Thank you for this opportunity to review this paper, which I greatly appreciated. | The authors thank Reviewer 2 for the encouraging and supportive review. We have revised the manuscript throughout to report on the societal impact of AOD abuse, both as reflected in the literature, and in the expressions and inputs of primarily community-based stakeholders during the process reported here. The paper has been supplemented with additional attention to the scale and extent of the AOD crisis in South Africa. We include further detail and particularities about the issue in the country. | 3-5  19-21 |
| Abstract: Really good abstract, with clear goals, methods, findings & nice recap of conclusions as separate to discussion & injecting timely reflections from vantage point of participatory action-learning facilitators. | Thank you. | - |
| Intro: Please provide some background to drug and alcohol abuse generally in South Africa and why, other than the misclassification of deaths due to drug/alcohol abuse. Statistics are provided for HIV, but then you apply the methods to a different health scourge altogether, and this seems like an oversight as it stands. LNS 440-445 provide this information, and while not inappropriate for discussion, feel like it would set the scene better in intro for researchers’ choice to focus on AOD for PAR & not other issue. | The Introduction section has been thoroughly revised to include more information on AOD abuse in South Africa, including its social drivers and impacts. | 3-5 |
| VAPAR, even though VAPAR)/Wits/Mpumalanga/Department of Health Learning Platform as an organizational collaborator on the research (as per title page), and approach explained on LN 136, there is still a disconnect between the reported causes of death and its application to alcohol & drug-related behaviors. This term is unfamiliar, even though it is a key word. Please make connect of VAPAR to PAR, as it remains unclear. | We have revised the Methods with a fuller description of VAPAR, the VA and PAR elements within this, and how they relate to one another. We also provide a fuller description of what PAR is, and provide a reference to a key text on the method. | 5 |
| LN 69 use of in-action: a play on words (i.e., inaction), or a subset like action-learning? Not being familiar with sector terminology, this term created confusion. | This term was drawn from the title of a paper the authors found insightful. Nevertheless, in the revised manuscript, we have used ‘inaction’ (no hyphen) and included the word ‘inertia’ in brackets to explain what is meant. | 3 |
| LN 82: HIV prevalence…missing the word IS in 1st part of sentence | Typographical error has been corrected. | 3 |
| LN 86: human resources crises related to pandemic, exacerbated by it, or previous to pandemic as well? Maybe return to this point & connect it to paragraph beginning LN 105. | At this point, we are describing the health system contexts generally, this does not relate to C-19. We have clarified this with the wording: ‘Serving the vast majority of the population, the public health system deals with this complex burden in the face of systemic underinvestment, human resource crises, corruption and deteriorating infrastructure’ And, at later point in the paper: ‘Sustainability challenges also relate to COVID-19 and the introduction of considerable, new demands in already-challenged systems.’ | 4, 21 |
| LN 153: policy implementation gaps—situate this statement in the context of official post-apartheid South Africa. Is health sector any better or worse than other sectors? And give some framing of why local community health delivery is more promising to close those policy gaps than other sectors. | The text has been edited to situate policy-implementation gaps in a post-apartheid context. And, to refer to these issues across sectors, as well as within health. Finally, the text has also been revised to set out why community health systems offer solutions to address these gaps. | 6 |
| Recruitment and snowballing of interviewees as study population grew is very well described. One place to expand is LN 172: what is population of village & size of regional area of local community-based health care ward? Having context to better understand the overall challenge you are stating would be well served by problem context. Literacy rates of community? Unemployment rates among young people affected by alcohol & drug abuse. Reader is left to conjecture causes of problem without facts. | The text has been edited to provide these details. | 6 |
| Another is at LN 174: How was recruitment of initial participants undertaken, by reputation, by nomination of another health organization? Please provide brief clarification as to how they were selected. LNs 183-184: same issue for next stage of nominations. | The text has been revised to provide an account of the purposive sampling, and the means through which participants were recruited. | 7 |
| Nice sequence of meeting steps under Step 2: Analyse/Plan. | Thank you for the comment | - |
| LN 206: need “on” at end of line | This typographical error has been corrected. | 8 |
| LN 234: copy of “structured tool” in supplementary materials would be helpful to see. | We have provided the tool; contained in supporting material in the revised paper. | 9 |
| LN 510: Local Action Plan: which stakeholders involved, at what levels of authority. This is necessary before  dissection of its drawbacks & potential challenges to implementation. | Thank you for this important comment. The manuscript has been revised to provide more detail both in the text and tables, on the specific levels and sections and the strategic and operational levels at which there was engagement with the process and commitment to progressing collective action. | 9 |
| LN 239: 16 follow-up visits to whom? At this point, it needs to be reestablished who your active participants are. Would be helpful to summarize prior to analysis how many total individuals overall participated in the process and contributed to your collected data? | The text has been revised to indicate more clearly who the participants were at this stage – those participants who had committed to leading on specific action items. We have also updated the methods section to make it clear how many participants were involved in each of the Steps 1-3. As noted below, we have opted to retain Figure 1, owing to comments from Reviewer 1, and because it helps summarise this information also, and is referred to in the revised text. | 9  5-11  Table 1  Fig. 1 |
| LNs 260 & 261: Structuration theory. Both lines support your public health research use of the theory for institutional-actor interrelations. But for those unfamiliar with this technical term, would be useful to define it here independently of the many citations. | The text has been revised to provide a fuller description of structuration theory. | 10 |
| Methods: did you use any qualitative data software or organizing system for thematic coding & analysis? Please specify software name if you did. | The team used NVIVO for thematic analysis and data management, the text has been revised to reflect this. | 10 |
| Some more knowledge of participant base: different ethnicities/groups represented, any division along lines of authority based on tribe-based financial status or privilege? In other words, was the arena for discussion “flat” in terms of power-bases? | The revised manuscript provides more details on the particulars of the participants, and considers the potential for power asymmetries throughout. | 5-11  13  22 |
| Similarly, at LN 523: South Africa’s COVID response was noteworthy, but in which direction? Did community-level implementation trump the competing challenges you mention, or did this impede success? Before moving on to future urgency, need to inform how successful (and therefore imitable) COVID community-level response was. | The manuscript has been revised to provide an account of how an initial highly centralised strategy to contain the virus and suppress transmission was effective. We also describe how the approach was rooted in an appreciation of the need for community-based health.  However, we further contextualise this in a short account of the following period of the pandemic in which a more holistic understanding of burden of disease that the health system is dealing with is provided, as well as a reality check on the underfunding and lack of recognition and other systemic threats to the CHW and WBPHCOT process.  We frame this account as necessitating of new forms of evidence based medicine, which are ongoing, committed to learning at local levels and build alliances together with service users and researchers. | 19, 21  23 |
| Need more quotes such as LN 565 by Joshi. Very informative to hear in first-person voice participant reactions & recognitions of reality behind social accountability and perceived effectiveness of citizen engagement. | Thank you for this comment. We have provided more quotes throughout the Results section to reflect the direct voice of study participants. | 11-18 |
| Methodological reflections are very strong, thoughtfully presented and grounded in research team’s long-term engagement and expertise in rural community health. While transferal to subnational and national contexts is raised, one would be curious as to applicability to extra-national contexts? Is success of the PAR approach contingent on civil society functionality? Thailand is mentioned, where trust is/was high; South Africa, one would suspect, less so, given histories of two contrasted countries. A note on generalizability in light of political cultures where PAR is being pursued is therefore merited. | Thank you for this insightful comment. We have revised the description in line with the contextual consideration to highlight that, while ‘context matters’, with critical constraining or enabling influences from social and political contexts, even in settings of deep division between communities and authorities and deeply hierarchical top-down operational contexts, learning processes can support new ways of collective learning and action. | 21 |
| Do not feel that figures 1 or 2 add to reader’s understanding of the process, perhaps due to the quality of the reproduction or the density of the text in these figures and suggest dropping them. The three tables are excellent in their organization and content, and the photos capturing participant interaction very descriptive without need of these additional visuals. I feel in the end, they draw away from the effectiveness of your other visuals the research team furnishes. | Thanks you for this comment. We have removed Figure 2. However, we have opted to retain Figure 1, considering especially Reviewer 1’s remarks on the need to understand the participant base, and how this relates to the process overall. However we have edited it to improve its reproduction. | Fig. 1 |

END OF RESPONSE TO REVIEW
