# Supplementary material for: ‘Voice needs teeth to have bite’! Expanding community-led multisectoral action-learning to address alcohol and drug abuse in rural South Africa
Source: PLOS Glob Public Health. 2022 Oct 19;2(10):e0000323. doi: 10.1371/journal.pgph.0000323 (PMC10022044; doi:10.1371/journal.pgph.0000323)
Supplement: Attachment — Submitted filename: Response to Review.docx [file pgph.0000323.s007.docx]

| Reviewer(s)' Comments to Author(s): | Author response | Page |
| --- | --- | --- |
| Reviewer #1: |  |  |
| I appreciate the opportunity to review this manuscript. I only have comments regarding the discussion.  I suggest restructuring this by pointing to the results obtained that are related to the participatory process (process forms and dynamics; actor interactions and interfaces; impacts and degrees of impacts; and enabling and constraining contextual factors) rather than the issue of AOD.  The way in which the discussion is currently structured seems confusing to me, opening spaces that until then had not been presented (such as COVID) and that may be part of the limitations of the study.  In particular, I suggest generating one full reflective argumentative paragraph about the level of achievement of the original plan and another that addresses this point "...giving the voice teeth. Achieving bite, however, requires longer-term engagement and more formal connections to the system." mainly because it happens to be the title of the manuscript. | We have reworked the Discussion to respond to this point. The revised Discussion opens with the section: ‘Transferrable learning’ (also made in response to comments from Reviewer 2). Here, we present a reflexive argument on the process with reference to the theory of change and analytical framework. This section includes reflections on levels of achievement of the action plan.  The revised Discussion then considers ‘achieving bite’ i.e., how the learning informs the reconstituting of spaces, structures, and processes to sustain the approach. Here, we reflect on what success is, and to whom, making that point that while the action plan was only partially achieved, the *process* was seen as acceptable and of practical benefit to those involved, supporting its uptake.  Regarding the content on the issue of AOD (and acknowledging feedback from Reviewer 2 throughout to provide a granular and grounded account of the AOD abuse context in South Africa), we have reworked this content into a second section ‘Relevance and practical utility’ to demonstrate that the process surfaced issues with both local and wider importance andac situating the contextual drivers supporting sustainability within the wider evidence base. | Discussion 17-22 |
| Reviewer #2: |  |  |
| Dear authors, Congratulations on returning such a strong revision of your important initial submission. You thoughtfully incorporated both reviewers’ comments and suggestions, making for a strongly grounded and fluently argued case study of VAPAR. The added introductory Detail of South Africa’s AOD’s landscape provides the necessary and compelling urgency of the problem you later to explore. Technical terms like VAPAR and structuralization are satisfactorily explained. Participant quotes are vivid and well-placed lending in an immediacy missing prior. Ver minor details to attend to which do not hold up my recommendation for publication, but please address: | Thank you for the supportive review and constructive comments, these are greatly appreciated and have vastly strengthened the paper, we are very grateful. | - |
| 1.line 377-78: missing the word are after “something you” | Amended | 13 |
| 2. Line 405: “otherwise… (Supplementary information)”. Something dropped | Thank you, amended with removal of ‘otherwise’ (sentence beginning ‘otherwise’ had been relocated earlier in same passage in the previous revision). | 14 |
| 3. Line 427: [The workshops] . Drop the it at beginning of sentence | Amended | 14 |
| 4. Line 486: some (xx). What you have in parentheses currently modifies some | We could not identify the term ‘some’ or content in parentheses at line 486. However, we have amended the passage beginning line 446, specifying the number of actions that relate to address this point. | 15 |
| 5. Line 510: eliminate colon after to | Amended | 17 |
| 6. Line 515: The section at end of results titled transferable learning seems more appropriate for the discussion section | This section of the results has been relocated into the Discussion section. | Discussion  17-22 |

END OF RESPONSE TO REVIEW
